# Relationship Between Force-Velocity Characteristics and Sprint Performance in Elite Sprinters: A Pilot Study

**DOI:** 10.7759/cureus.65944

**Published:** 2024-08-01

**Authors:** Ayrton Moiroux--Sahraoui, Jean Mazeas, Jaabir Mohammad Jhingoor, Maurice Douryang, Ismail Bouzerkraoui Alaoui, Florian Forelli

**Affiliations:** 1 Sports Rehabilitation, Orthosport Rehab Center, Domont, FRA; 2 Orthopaedic Surgery, Clinic of Domont Ramsay Healthcare, Domont, FRA; 3 Physiotherapy and Physical Medicine, University of Dschang, Dschang, CMR; 4 Research Unit, Mohammed VI Center for Research and Innovation, Rabat, MAR; 5 Faculty of Nursing and Allied Health Professions, Mohammed VI University of Sciences and Health, Casablanca, MAR; 6 Research Unit, Société Française des Masseurs Kinésithérapeutes du Sport (SFMKS) Lab, Pierrefite sur Seine, FRA

**Keywords:** elite sprinters, biomechanical variables, rate of torque development, hamstrings, sprint

## Abstract

Background: Sprinting is a type of running that consists of producing a short and intense effort in order to perform maximal speed in a short period of time. Sprinting is widely investigated because of the multiple and complex mechanisms involved. Sprint studies are now focusing on how to improve performance by focusing their analyses on the mechanic variables. The hamstrings are part of the most important muscle groups during sprinting because of their role of stabilization and propulsion, but they must be able to produce the maximum strength during a short moment, this ability is characterized by rate of torque development (RTD). The main of this study was to investigate the association between hamstring RTD (Nm.s^-1^.kg-^1^) and mechanical variables composed of maximal power output (Pmax) (W.kg^-1^), maximal theoretical velocity (V0) (m.s^-1^), and maximal horizontal force production (F0) (N.kg^-1^) on short sprints in elite sprinters.

Methods: For this clinical trial, we used a research method based on data collection. A single group composed of four male and one female elite sprinters (age: 17.2 ± 1.79 years) has been included in this study. The sprinters performed a hamstring strength test, which included five trials of four seconds for each leg. This test required a portable dynamometer, the Kforce®, to collect 100 and 200 milliseconds RTD. Then the subjects were submitted to a sprint test. My Sprint® application has been used to collect the sprint mechanical variables. The sprint test included two trials; a starting block has been used for this test. A linear regression analysis was used between the rates of torque development and mechanical variables composed of maximal power output (Pmax) (W.kg^-1^), maximal theoretical velocity (V0) (m. s^-1^), and maximal horizontal force production (F0) (N.kg^-1^).

Results: No significant correlation was observed between 100 and 200 ms hamstring RTD and sprint biomechanical variables: maximal power output (Pmax) (W.kg^-1^), maximal theoretical velocity (V0) (m. s^-1^), and horizontal force production (F0) (N.kg-1). However, 100 and 200 ms RTD collected on the dominant leg tends to be more correlated with maximal theoretical velocity (spearman’s rho = 0.80; p-value = 0.13) and Pmax output (spearman’s rho = 0.70; p-value = 0.23) while for non-dominant leg, RTD tends to be more correlated with maximal power output (spearman’s rho = 0.60; p-value = 0.35) and horizontal force production (spearman’s rho = 0.70; p-value = 0.23).

Conclusions: Hamstring RTD is not correlated with sprint biomechanical variables in elite sprinters. Further investigations must be made to study the observed trends in this study.

## Introduction

Hamstring strain injuries are among the most common non-contact injuries with short- and long-term consequences in athletes [1]. Studies have found that most hamstring injuries occur during running or sprinting [2]. Male athletes seem to be more affected by hamstring injuries than females [2,3].

Among the various causes of hamstring injuries, there are modifiable risk factors such as weak hamstrings, weak hip extensors, and fatigue [4], imbalance between eccentric hamstring torque and concentric quadriceps force [5], explosive strength deficit [6], deficient extensibility of the quadriceps [7], and hip flexors [6-8] associated with uncontrolled anteversion of the pelvis during sprinting [9].

The force/velocity ratio is described in studies by the term "rate of torque development" (RTD). This consists of analyzing the maximum contraction force that the muscle can produce over a given period [10]. Scientific research has shown that the force/velocity ratio is directly linked to sporting performance in jumping [11-13], cycling [14], and golf swinging [15].

Ground reaction force has been shown to be a key characteristic of sprint performance [11]. One study showed a significant link between the production of a high force/velocity ratio (RTD) and the production of a high ground reaction force, demonstrating that there is a correlation between force/velocity ratio and sprint performance [12].

Several studies have looked at the analysis of important determinants of sprinting. Among these different determinants, we find mechanical parameters [16]. These variables include horizontal force (F0) (N.kg-1), maximum velocity developed (V0) (m.s-1), maximum horizontal power relative to body mass (P max) (W.kg-1), and the DRF (application/direction force index), which represents the athlete's ability to maintain horizontal force production while speed increases during sprinting [17]. The shorter the distance, the greater the horizontal force (F0). Maximum developed speed (V0) is strongly correlated with sprint performance over 10m and, as distance increases, the correlation becomes stronger [18]. Maximum power showed a very strong correlation with sprint performance. However, the improvement in sprint time did not have a linear relationship with the increase in maximum power relative to body mass. In fact, it has been calculated that a 5% increase in speed would require a 16% increase in maximum power relative to body mass, indicating a nonlinear relationship [19].

The primary objective of this study was to investigate the relationship between the hamstring force/velocity ratio and the mechanical variables of force, velocity, and maximum power developed over short sprints in elite sprinters.

## Materials and methods

Study design

For this data collection research, a file was filed with the CNIL, bearing the number 225099. Subjects participating in the study were informed of the protocol's process through an explanatory document acting as informed consent after signature. Data obtained during the study are anonymous. The examinations carried out were free, non-invasive, and non-hazardous for the subject. The data collection method used was prospective. Data were collected between February 28, 2022, and March 15, 2022.

Participants

We evaluated eight subjects aged 17.2 ± 1.79 years, all high-level sprinters (60m and 100m runner) with a weekly training schedule of 10 hours. Athletes were included if they were aged between 16 and 20; were sprinters with a weekly activity > 8 hours; were able to perform sprints; and had an activity level greater than or equal to 8 on the Tegner scale.

The general exclusion criteria were subjects who had suffered a lower limb injury in the previous 6 months and those with pain or discomfort sufficient to influence their ability to sprint or perform any of the study exercises. Subjects with lower limb pain greater than 20 on VAS were also excluded from the study. In addition, subjects taking part in the study should not have cognitive and/or neurological disorders. 

The subjects represent a population of athletes aged between 16 and 20 who can perform a pain-free sprint. Patients’ baseline information was recorded, and demographics (age, size, weight, body mass, sex).

Blinding

Subjects participating in the study were not informed of the study's hypotheses and data. No feedback on values or protocol was given to patients. However, it is impossible to rule out the possibility that they had contact with each other outside the sessions in which the data were measured.

Outcome measure 

Hamstring isometric strength was measured by the KForce Muscle Controller dynamometer (Kinvent, Montpellier, France). It can measure forces up to a maximum of 90kg and has a 150mAh Li-Po battery that provides 5h00 of autonomy. It transmits its data on a 2.4GHz band with a range of up to 10 meters. Data is transmitted via Bluetooth connection and obtained in real time via KAPA INVENT's Kforce® mobile application. When the muscle strength test was carried out, the Kforce® application was running version 5.3.0.

The computer used was an Asus® UX301LAB, for statistical processing of the data collected during the various tests. To measure the various mechanical determinants of interest to our study, we used the My Sprint® application download on an iPad Air® (4th generation).

Data collection focused on the correlation measured between independent predictors of hamstring muscle function and dependent predictors of sprint performance. The independent variables are peak hamstring force torque (Nm.kg-1), and hamstring force/velocity ratio over the 0-100 ms interval. (RTD100 in Nm.s-1.kg-1) hamstring force/velocity ratio over the 0-200 ms interval (RTD200 in Nm.s-1.kg-1). The dependent variables are horizontal force F0 (N.kg-1) maximum developed velocity V0 (m.s-1), and maximum horizontal power relative to body mass P max (W.kg-1).

Intervention

Our protocol is based on the study by Morin et al. [20]. Each study subject completed a written consent form before participating in the study. Each subject was interviewed separately. The following information was collected: weight, height, sex, date of birth, history of lower-limb injuries, dominant limb. This information was then saved in an Excel® spreadsheet. All data (RTD, VO, F0,P max) measured during the various exercises were randomized. Study participants performed the hamstring strength test first, followed by the sprint test. 

When taking dynamometric measurements of hamstring strength, the dynamometer is set up to be connected via Bluetooth to the Kforce® mobile application, so that data can be collected in real time. The subject lies on his stomach, with the leg being tested at 15° of knee flexion. We ask subjects to stabilize themselves by grasping the sides of the table with their hands for the duration of the test.

To minimize hip flexion during the test, a rigid strap is placed around the pelvis. The dynamometer is placed on the posterior surface of the ankle, 5 cm from the ankle's lateral malleolus. Following two training trials, three more are performed, each separated by 60 seconds of rest. The subject is instructed to release the leg completely before each trial to relax muscles. The examiner encourages each subject "You're ready-3-2-1-Go-Push-Push-Release". Each leg is tested separately, and only the best attempt of the three trials is retained. The data collected via the mobile application is then extracted into an Excel® spreadsheet. All measurements are then normalized to the body weight of each subject.

For this sprint test, we used the My Sprint® application. The tablet is placed on a tripod 18 meters from the stake placed 15 meters away, the other stakes are placed 5.57 meters, 10.28 meters, 19.72 meters, 24.43 meters, and 29.15 meters away. The subject performs two sprints, after a warm-up, at maximum speed, with a 3 minutes' rest, using a starting block. The examiner gives the instruction "3-2-1-Start". The best of the two sprints is selected. Beforehand, the examiner videotapes the two sprints in slow motion at 240 frames per second. The various data are collected and then transferred to an Excel® spreadsheet.

Statistical analysis

We performed a flat sort of the Excel® data. The various variables studied do not follow the normal distribution. The level of significance was set at 0.05. First, we performed descriptive statistics with the mean and standard deviation on the age, height, weight, and gender of our test group.

In order to study the correlation between the force/velocity ratios: RTD100 (Nm.s-1.kg-1) (over 100 milliseconds); RTD200 (Nm.s-1.kg-1 ) (over 200 milliseconds) and the biomechanical determinants of sprinting, namely, V0 (maximum developed speed in m.s-1); F0 (horizontal force in N.kg-1); P max (maximum horizontal power relative to body mass in W.Kg-1 ), we chose to perform Spearman's correlation test. Statistical analyses were performed using JASP® software (version 0.16.1.0).

## Results

Demographic and baseline characteristics

The mean age of the participants was 17.20 ± 1.79 years. A summary of baseline data (age, BMI, gender, weight, and size) is presented in Table 1. We had previously selected eight subjects to participate and three of these eight subjects had to be excluded from the study. These three subjects were excluded by decision of the technical staff (do not follow instructions correctly or because the data collected were not usable.

**Table 1:** Demographic and baseline characteristics Note: values are presented as means ± standard deviation unless stated otherwise. y: Years; m: meters; kg: kilograms; M/F: male/female ratio; cm: centimeters; R: right; L: left

Baseline characteristics	Test group (n = 5)
Age (y)	17.2 ± 1.79
Body mass index (kg/m^2^)	21.6 ± 1.8
Sex (M/F)	4 / 1
Size (cm)	176.6 ± 10
Weight (kg)	67.6 ± 11.8
Dominant size (R/L)	5/0

Calculation of correlation between the force/velocity ratio over 100ms (dominant leg) and sprint biomechanical data

Statistical analysis reveals that RTD100 correlates well with F0 (spearman rho = 0.40), V0 (spearman rho = 0.80), and P max (spearman rho = 0.70). However, the correlations observed between RTD100 and V0 (p-value = 0.52), between RTD100 and F0 (p-value = 0.13), and between RTD100 and P max (p-value = 0.23) were not significant (Table 2).

**Table 2:** Results of Spearman's correlation test between the force/velocity ratio at 100ms measured on the dominant leg and the various mechanical variables of sprinting Nm: Newton meter; kg: kilogram; s: second; m: meter; W: watt

Ratio	Variables	Spearman’s Rho	p-value
RTD100 (Nm.s-1.kg-1)	- F_0 _(N.kg^-1^)	0.400	0.517
RTD100 (Nm.s-1.kg-1)	- V_0 _(m.s^-1^)	0.800	0.133
RTD100 (Nm.s-1.kg-1)	- P max (W.kg^-1^)	0.700	0.233

Calculation of correlation between the force/velocity ratio over 200ms (dominant leg) and sprint biomechanical data

Statistical analysis reveals that RTD200 correlates well with F0 (spearman rho = 0.40), V0 (spearman rho = 0.80), and P max (spearman rho = 0.70). However, the correlations observed between RTD200 and V0 (p-value = 0.52), between RTD100 and F0 (p-value = 0.13), and between RTD200 and P max (p-value = 0.23) were not significant (Table 3).

**Table 3:** Results of Spearman's correlation test between the force/velocity ratio at 200ms measured on the dominant leg and various mechanical sprint variables Nm: Newton meter; kg: kilogram; s: second; m: meter; W: watt

Ratio	Variables	Spearman’s Rho	p-value
RTD200 (Nm.s-1.kg-1)	- F_0 _(N.kg^-1^)	0.400	0.517
RTD200 (Nm.s-1.kg-1)	- V_0 _(m.s^-1^)	0.800	0.133
RTD200 (Nm.s-1.kg-1)	- P max (W.kg^-1^)	0.700	0.233

Calculation of correlation between the force/velocity ratio over 100ms (non-dominant leg) and sprint biomechanical data

Statistical analysis reveals that RTD100 correlates well with F0 (spearman rho = 0.70), V0 (spearman rho = -0.10), and P max (spearman rho = 0.60). Nevertheless, the correlations observed between RTD100 and V0 (p-value = 0.23), between RTD100 and F0 (p-value = 0.95), and between RTD100 and P max (p-value = 0.35) were not significant (Table 4).

**Table 4:** Results of Spearman's correlation test between the force/velocity ratio at 100ms measured on the non-dominant leg and the various mechanical sprint variables Nm: Newton meter; kg: kilogram; s: second; m: meter; W: watt

Ratio	Variables	Spearman’s Rho	p-value
RTD100 (Nm.s-1.kg-1)	- F_0 _(N.kg^-1^)	0.700	0.233
RTD100 (Nm.s-1.kg-1)	- V_0 _(m.s^-1^)	-0.100	0.950
RTD100 (Nm.s-1.kg-1)	- P max (W.kg^-1^)	0.600	0.350

Calculation of correlation between the force/velocity ratio over 200ms (non-dominant leg) and sprint biomechanical data

Statistical analysis reveals that RTD200 correlates well with F0 (spearman rho = 0.70), V0 (spearman rho = -0.10), and P max (spearman rho = 0.60). Nevertheless, the correlations observed between RTD200 and V0 (p-value = 0.23), between RTD200 and F0 (p-value = 0.95), and between RTD200 and P max (p-value = 0.35) were not significant (Table 5).

**Table 5:** Results of Spearman's correlation test between the force/velocity ratio at 200ms measured on the non-dominant leg and the various mechanical sprint variables Nm: Newton meter; kg: kilogram; s: second; m: meter; W: watt

Ratio	Variables	Spearman’s Rho	p-value
RTD200 (Nm.s-1.kg-1)	- F_0 _(N.kg^-1^)	0.700	0.233
RTD200 (Nm.s-1.kg-1)	- V_0 _(m.s^-1^)	-0.100	0.950
RTD200 (Nm.s-1.kg-1)	- P max (W.kg^-1^)	0.600	0.350

## Discussion

This study demonstrated that hamstring RTD is not correlated with sprint biomechanical variables. According to the study by Ishøi et al. [21], there is a correlation between the 200 ms hamstring force/velocity ratio and horizontal force (F0) (described according to a medium association strength) and a correlation between the 100 ms hamstring force/velocity ratio and maximum power developed (P max) (described according to a medium association strength). If we compare these results with our present study, we find results in the same direction: in our case, horizontal force correlates well with the force/velocity ratio over 200ms, but also over 100ms (spearman's rho = 0.4) measured on the subjects' non-dominant leg. We also found the same results regarding the correlation observed between maximum power developed (P max) and the force/velocity ratio over 100ms of the hamstrings observed in the study by Ishøi et al. [21] and in our study. In our case, we also found a correlation between the force/velocity ratios at 100 and 200 ms and the maximum power developed for both the dominant leg (spearman's rho = 0.7) and the non-dominant leg (spearman's rho = 0.6) of the subjects.

According to the study by Morin et al. [17], there is indeed a significant correlation between horizontal force (F0), during the acceleration phase of sprinting (more precisely at the end of the oscillation phase), and the peak of the eccentric torque, and a significant trend towards correlation was found when considering the peak of the concentric torque, of the lateral knee flexors (more precisely the biceps femoris). If we compare these results with those of our study, they both seem to point in the same direction, as we also observe a strong correlation between horizontal force and force/velocity ratios at 100 and 200 ms (spearman's rho = 0.7) measured on the subjects' non-dominant leg. However, Morin et al. chose an isokinetic machine to perform their tests, whereas in our study we chose a portable dynamometer.

Tillin et al. [22] found strong inverse correlations between the onset phase (0-100 ms) of isometric explosive squats and time in 0-5-meter and 0-20-meter sprints, meaning that high values for explosive squats were correlated with fast times in 0-5-meter and 0-20-meter sprints. This study clearly demonstrates that the ability to produce a high level of force over a short period of time (defined by the force/velocity ratio) appears to be an important factor in sprint performance. When we compare the results of the study by Tillin et al. with our own, we can see a certain similarity in that we observe a negative correlation (weak but existing) between the force/velocity ratios at 100 and 200ms measured on the subjects' non-dominant leg and the maximum speed developed (spearman's rho = -0.1), which would be in line with the inverse correlations observed by Tillin et al. Nevertheless, in our study we observed a very strong positive correlation between force/velocity ratios at 100 and 200ms measured on the subjects' dominant leg and maximum velocity developed (spearman's rho = 0.8).

Finally, as mentioned above, none of the results of our study is significant about the correlation between the force/velocity ratio and the various biomechanical determinants of the sprinting, whereas the results of these studies are significant.

Limitations

The study has several limitations that may affect the results and conclusions. Further investigations with larger sample sizes are recommended. Participants were recruited from a rehabilitation center. Despite this diversity, the results were significant and applicable to a variety of patient profiles. Indeed, the subjects who had not yet performed their tests were obliged to continue their training sessions, so we can't rule out the possibility that fatigue may have influenced the results, either positively or negatively.

Furthermore, no randomization was carried out to determine the order in which the tests were performed. Participants in our study always took part in the hamstring strength test first, followed by the sprint test. Possible measurement bias may be due to the equipment or procedures used. 

## Conclusions

The results of the statistical analysis revealed positive correlations between RTD100 measured on the dominant leg and the mechanical sprint variables: F0, V0, and Pmax. The same results were observed for RTD200 on the right leg. However, these correlations are not significant. For the non-dominant leg, RTD100 shows positive correlations with F0 and Pmax, but a negative correlation with V0. The same trends were observed for RTD200 on the left leg. Here again, correlations are not significant.

Although correlations between RTD (100 and 200) and mechanical sprint variables (F0, V0, Pmax) are observed, their non-significance leads us to reject the study's initial hypothesis. This means that there is insufficient evidence to support a relationship between the hamstring strength/speed ratio and mechanical determinants of sprint performance. At present, there are very few studies looking solely at the correlation between the force/velocity ratio of the hamstrings alone and the biomechanical determinants of sprinting, since most of the time it is force couples between the quadriceps and hamstrings that are considered. Future investigations involving only the hamstrings need to be carried out to better understand the role and importance of this muscle group in sprinting.
